# Adenosine triphosphate overrides the aversive effect of antifeedants and toxicants: a model alternative phagostimulant for sugar-based vector control tools

**DOI:** 10.1186/s13071-023-06039-x

**Published:** 2023-11-14

**Authors:** Matthew Lukenge, Rickard Ignell, Sharon Rose Hill

**Affiliations:** Disease Vector Group, Unit of Chemical Ecology, Department of Plant Protection Biology, Swedish University of Agricultural Sciences, Alnarp, Sweden

**Keywords:** Adenosine triphosphate, Taste, Toxins, *Aedes aegypti*, Feeding, Mosquito, Attractive toxic sugar bait

## Abstract

**Background:**

Sugar, when used as the phagostimulant in attractive toxic bait control tools, limits the efficacy and selectivity of this technology. Thus, more potent and selective phagostimulants than sugar are required to improve this technology. The potency of adenosine triphosphate (ATP) as an alternative model phagostimulant was assessed to determine its capacity to override the aversive effects of select antifeedants and toxicants. How ATP and sucrose modulate the rate of toxicity in the yellow fever mosquito *Aedes aegypti* was also examined.

**Methods:**

A no-choice feeding assay was used to investigate the phagostimulatory ability of ATP to override the aversive effects of structurally divergent antifeedant and toxicant compounds, and to modulate the rate of toxicity over 24 h. Binary combinations of antifeedant and toxicant compounds, at various concentrations, were similarly assessed for enhanced lethal potency. In comparison, no-choice open access and cotton wick feeding assays were used to determine the phagostimulatory role of sucrose in the ingestion of boric acid-laced diets. Dissections of the guts were performed to determine the diet destination as dependant on the phagostimulant.

**Results:**

ATP is a potent phagostimulant that dose dependently overrides aversion to antifeedant and toxicant tastants. Feeding on antifeedant- or toxicant-laced diets that was induced by ATP selectively resulted in rapid knockdown (nicotine, lobeline and caffeine) or death (boric acid and propylene glycol), with a combination of the two lethal compounds inducing a synergistic effect at lower concentrations. ATP- and sucrose-induced feeding predominantly directed the antifeedant- or toxicant-laced meals to the midgut and the crop, respectively.

**Conclusions:**

ATP is an efficacious alternative model phagostimulant to sucrose that overrides the aversive effects of antifeedants and toxicants, resulting in rapid toxic effects. Furthermore, this study demonstrates that variation in the rate of toxicity between ATP- and sugar-induced feeding is at least partly regulated by the differential feeding response, volume imbibed and the destination of the meals. Additional research is needed to identify structurally related, stable analogues of ATP due to the ephemeral nature of this molecule. For future applications, the workflow presented in this study may be used to evaluate such analogues for their suitability for use in attractive bait stations designed to target a broad range of haematophagous arthropods and prevent off-target species’ feeding.

**Graphical Abstract:**

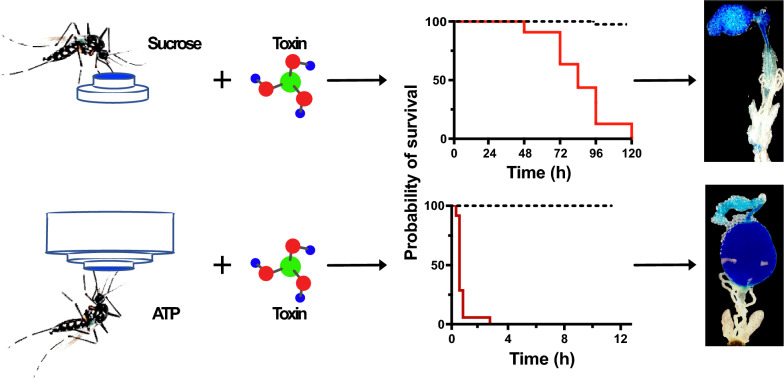

**Supplementary Information:**

The online version contains supplementary material available at 10.1186/s13071-023-06039-x.

## Background

Interventions targeting arthropod vectors are the most efficient strategies in the fight against vector-borne diseases [1]. With a limited arsenal of vector control tools, and those that are available becoming less effective [1, 2], the World Health Organization advocates for the development of new tools with different modes of action to be used in future integrated vector management strategies [3]. Although taste-based arthropod control tools date back as far as the eighteenth century [4], the model taste-based tool, the attractive toxic sugar bait (ATSB), has received insufficient attention. Improvements in ATSB efficiency and efficacy offer a viable avenue to increase their usefulness for integrated vector management strategies.

Current ATSB technologies have several limitations, primary among which is the use of sucrose as a non-selective phagostimulant, targeting select disease vectors and beneficial insects, e.g. pollinators, alike [5]. In addition, sucrose may not be an efficient phagostimulant to override the aversive effects of natural antifeedants and synthetic toxicants, as reliance on sugar phagostimulants is prone to resistance [6, 7]. Moreover, many disease vectors are obligate blood feeders that do not imbibe sugar meals [8], and are thus not targeted by ATSBs. For mosquito vectors, which tend to imbibe small sugar meals at a time, it may also require multiple visits to an ATSB station to accumulate a sufficient level of a toxin for it to be lethal. Thus, to mitigate the limitations highlighted, a more potent and selective phagostimulant, to which the cost of evolutionary pressure is too high for the vector to develop resistance, needs to be identified.

A model alternative phagostimulant for this purpose is adenosine triphosphate (ATP), a highly potent and strongly selective phagostimulant for the majority of blood-feeding arthropods [9]. The likelihood of blood-feeding vectors developing resistance to ATP is low because ATP is an indicator of a blood meal, which is required to secure fitness-related behaviours. Due to the labile nature of ATP [10], it cannot be used in current ATSB technology. An increased understanding of the signalling pathway and how this modulates the uptake of toxic antifeedants may, however, provide guidance for the identification and/or development of stable ATP analogues for future use in attractive toxic ATP-analogue bait technology. In this study, ATP was assessed for its ability to drive the feeding of the yellow fever mosquito *Aedes aegypti* on a variety of structurally different antifeedant and toxicant compounds that elicited varying degrees of toxicity, ranging from knockdown to lethal effects. Depending on the phagostimulant used, i.e. ATP or sugar, the diet destination differed, correlating with different rates of action of the toxic compounds. We discuss the use of ATP as a model for alternative phagostimulants to sugar as a driver for toxic baits in vector control tools.

## Methods

### Mosquito rearing

*Aedes aegypti* (Rockefeller strain) was reared at 25 ± 2 °C, 70 ± 2% relative humidity and a 12 h:12 h light:dark photoperiod. Briefly, eggs were hatched in plastic trays (23.5 cm × 18 cm × 7.5 cm; ca. 300 ml water), with ca. 300 larvae per tray. The pupae, which were collected in small plastic cups (30 ml water), were transferred into BugDorm-4E1515 cages (17.5 cm × 17.5 cm × 17.5 cm; Megaview Science, Taichung, Taiwan). Emerging adults were provided with ad libitum access to 10% weight/volume (w/v) sucrose up to 4 days post-eclosion, and then starved for 22 ± 2 h with ad libitum access to water prior to the feeding experiments.

### Bioassay to assess ATP-induced feeding on antifeedant and toxicant compounds

To make the feeding test solutions, stock solutions of 12 mM ATP [Chemical Abstracts Service (CAS) no. 34369-07-8; Merck, Darmstadt, Germany] were prepared by dissolving ATP in bicarbonate buffered saline [150 mM sodium chloride (Merck) and 10 mM sodium bicarbonate (Merck)] at pH 7.4 ± 0.07, and then stored at − 20 °C. To examine whether ATP overrides feeding deterrence and induces feeding on natural antifeedants or synthetic toxicants, a panel of tastants, previously classified as antifeedants, toxicants or feeding deterrents, were assessed at a range of concentrations (Table 1). The natural antifeedants were the plant-derived alkaloids caffeine [11, 12], quinine, nicotine [11, 13, 14], lobeline [13] and capsaicin [15, 16], while the toxicants were the synthetic compounds *N*,*N*-diethyl-meta-toluamide (DEET), a major insect repellent [13], and propylene glycol [17], and the mineral acid boric acid, which are known insecticides [16, 18–20] (Table 1). Stock solutions of the tastants were prepared as follows: water-soluble compounds, i.e. caffeine (80 mM), propylene glycol (10.5 M), DEET (209 mM), nicotine (20 mM) and boric acid (1.3 M), were dissolved in the bicarbonate buffered saline. This was done at room temperature, except for boric acid and quinine that required heating at 42 °C to fully dissolve. In contrast, lobeline hydrochloride (100 mM) and capsaicin (100 mM) were dissolved in 50% (volume/volume; v/v) ethanol, while quinine (20 mM) was dissolved in 30% (v/v) ethanol. Working dilutions were prepared for lobeline hydrochloride and capsaicin at 5 mM, and for quinine at 2 mM in the bicarbonate buffered saline. The final concentrations contained less than 2.5% (v/v) ethanol. Ethanol at 2.5% (v/v) was examined for its effect on ATP phagostimulation in a membrane feeding assay, as described below, which demonstrated that it had no effect on ATP potency [*U*_(9)_ = 10.50, *Z* = 0.30, *P* > 0.99].

**Table 1:** Antifeedant and toxicant compounds and concentration ranges evaluated in the presence of 0.6 mM adenosine triphosphate (ATP)

Compound	CAS number	Concentration (mM)
Caffeine	58-08-02	0.10–20
Nicotine	54-11-5	0.10–3
Quinine	130-95-0	0.0010–2
Lobeline hydrochloride	134-063-4	0.10–3
Capsaicin	10,045-35-3	0.10–5
Boric acid	57-55-6	1.60–323.50
Propylene glycol	134-62-3	131–2628.50
DEET	404-86-4	0.0050–53.30

*DEET* *N*,*N*-diethyl-meta-toluamide,* CAS* Chemical Abstracts Service

Each stock solution of the tastant compounds was serially diluted using the pH-controlled bicarbonate buffered saline to which was added an equal volume of ATP to a final concentration of 0.6 mM, a concentration that elicited maximum phagostimulation [maximal effective concentration (EC_max_)] in *Ae. aegypti*, as determined in a preliminary analysis, through the generation of a dose–response curve with nine doses between 0.005 and 0.6 mM (data not shown), and supported by a previous study [21]. In addition, xylene cyanol FF (CAS no. 2650-17-1; Merck) was added to a final concentration of 1 mg ml^−1^ to aid visualisation of engorged individuals. The final concentration ranges of the tastants are indicated in Table 1. A positive control (0.6 mM ATP in buffer) and two negative controls, i.e. buffer alone and buffer together with the lowest concentration of each antifeedant and toxicant, were used, in which avid probing, but no engorgement, was observed.

To confirm whether the mosquitoes detected the antifeedants and toxicants, a concentration at, or close to, the threshold of behavioural response was used. The assay included a negative control (antifeedant or toxicant “tastant” alone) and two positive ATP controls, the 50% and fully effective concentrations (respectively, EC_50_ 0.072 mM and EC_max_ 0.6 mM, as determined in preliminary studies; see above), as well as the tests, i.e. combinations of the tastant with either of the ATP concentrations (*n* = 5, *n* = 50).

**Fig. 1a–c:**
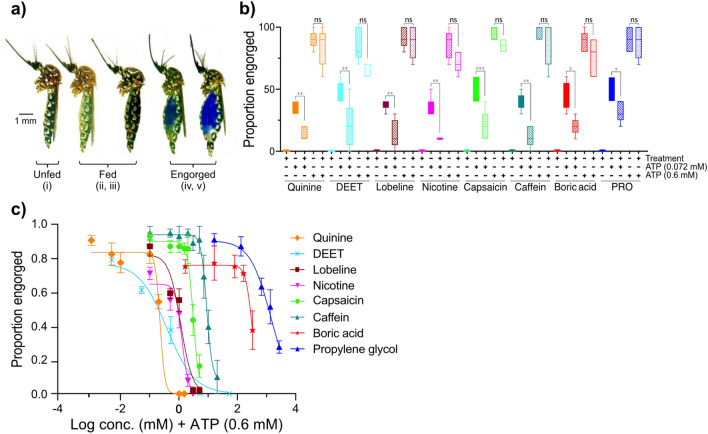
Adenosine triphosphate (*ATP*) overrides the aversive response to antifeedants and toxicants in *Aedes aegypti*. **a** The scale (*i*–*v*) used to score unfed to engorged individuals. **b** The proportion of *Ae. aegypti* engorged on meals of antifeedant and toxicant compounds, in combination with ATP at half maximal effective concentration (EC_50_) (0.072 mM) and EC_max_ (0.6 mM) (*n* = 50). The behavioural response of the mosquitoes to the antifeedant and toxicant compounds is aversive when these are presented in combination with ATP at EC_50_, whereas ATP EC_max_ is able to override this effect. ANOVA was used for the pairwise comparisons; *ns* non-significant, * *P* < 0.05, ** *P* < 0.01. **c** Mosquitoes engorged on 0.6 mM ATP-containing antifeedant and toxicant diets in a dose-dependent manner. The error bars indicate the SEM. The number of replicates for each diet and concentration was 50.* DEET* *N*,*N*-diethyl-meta-toluamide,* PRO* propylene glycol

Ten female mosquitoes at 5 days post-eclosion were gently aspirated into bioassay chambers (tall polystyrene Petri dishes, 12 cm diameter × 6 cm height; Semadeni, Ostermundigen, Switzerland) covered with fine mesh, for each of the controls and tests, and for five replicates. Using a membrane feeding system (Hemotek, Blackburn, UK), and feeding reservoirs (0.3 ml; Hemotek) filled with 200 μl of the diets under a collagen membrane (Hemotek), the mosquitoes were exposed to the diets for 30 min at 37 °C. Since *Ae. aegypti* is a diurnal feeder, the assays were performed at Zeitgeber time 6–9, within the peak activity period [22]. Mosquitoes that scored iv or v on the feeding scale were considered engorged, while those that scored above ii were considered fed (Fig. 1a), and used as such in further analyses.

### Toxic effects of antifeedant and toxicant diets imbibed during ATP-induced feeding

To examine the toxic effect of the antifeedant and toxicant compounds, a no-choice feeding assay was used as described above in which 10 mosquitoes were exposed to the antifeedant and toxicant diets and observed for 24 h without access to sugar and water. This experiment was replicated 5 times. Unlike in previously conducted sugar-bait toxic assays, in which the mosquitoes had access to the toxic diet ad libitum for the duration of the experiment [19, 20, 23], the mosquitoes in this experiment had access to the ATP diet for 30 min, since ATP is thermolabile and rapidly loses its integrity. Lethal and knockdown effects were scored after exposure to the diets for up to 24 h.

### Toxicity of sucrose-induced feeding on boric acid

Taste-based toxic baits commonly include sucrose as a feeding stimulant. To compare our results of ATP-induced feeding on boric acid by *Ae. aegypti* to those of previous studies, an assessment of the sugar-induced feeding [10% (w/v) sucrose] on a boric acid diet was conducted. For this purpose, 10 female mosquitoes (5 days post-eclosion) were placed in a BugDorm cage and then exposed to the diet via either of two methods: a cotton wick soaked in a 1% (w/v) boric acid, 10% (w/v) sucrose (Merck) and xylene cyanol (1 mg ml^−1^) diet contained in a 5-ml glass vial; or an open-access feeding assay, in which 200 μl of the same solution was added to a Hemotek feeding reservoir (without a membrane), as described above, with refills every 20–24 h. In both experiments, eight replicates of the treatments and two replicates of the control [10% (v/v) sucrose plus dye (xylene cyanol)] were conducted. The survival of the exposed individuals was scored for up to 5 days.

### Synergistic effect of ATP-induced feeding on combined antifeedant and toxicant diets

In our panel of toxicants, boric acid and propylene glycol elicited lethal effects, while the antifeedants nicotine, lobeline and caffeine induced knockdown followed by recovery. We hypothesised that a combination of lower concentrations of each of the compounds would result in a synergistic lethal effect. To address this hypothesis, the same membrane feeding assay as described above was performed with 10 female mosquitoes, and was replicated 5 times, using a combination of boric acid and propylene glycol, nicotine or caffeine (Additional file 1: Table S1). In all cases, the mosquitoes were stimulated to feed using 0.6 mM ATP, which also served as the positive control.

### Destination of ATP- and sucrose-induced diets

Having observed that the rate of toxicity in ATP-induced feeding was, by far, much faster when compared to sucrose-induced feeding, we hypothesised that this difference was in part due to the destination of the meals in either instance. To test this hypothesis, the lowest concentrations that induced the highest level of engorgement while still eliciting a toxic effect were considered for each individual compound. In brief, for the ATP-induced meal, the membrane feeding assay, as described above, was used. For comparison, a sucrose-induced feeding assay was used, in which cotton balls soaked with the diet were placed on top of bioassay chambers (Semadeni) and heated to 37 °C for 40 min. Each BugDorm contained 10 mosquitoes per diet. After exposure, the mosquitoes were anaesthetised on ice and their guts dissected in Ringer’s solution under a stereo microscope (×10, Nikon SMZ100; Nikon, Stockholm, Sweden) equipped with a 64 MP Android camera (Tecno Camon 19 Pro; Tecno, Shenzhen, China). The experiment was repeated until the guts of 10 fed individuals per diet had been successfully dissected.

### Statistical analysis

To test whether the mosquitoes detected the antifeedant and toxicant compounds, a one-way ANOVA was used to test the proportion of individuals that engorged on ATP (EC_50_) and those that fed on a combination of ATP (EC_50_) plus tastant. In addition, an ANOVA was used to test whether ATP (EC_max_) is able to override the aversive effect of the antifeedants and toxicants (ATP at EC_max_ plus tastant). To analyse the level of feeding aversion of mosquitoes across the antifeedant and toxicant compounds in the presence of ATP (EC_max_ 0.6 mM), the dose–response curves were compared using the least squares regression method in a non-linear regression model, in which the proportion of engorged mosquitoes was the response variable, while the diets were the predictor variables. An extra sum-of-squares* F*-test was used to compare whether the best fit values of select parameters (log EC_50_, Hill slope, top value) differed between individual antifeedant and toxicant datasets. To assess the mortality rates of both ATP-induced (24 h) and sucrose-induced (5 days) toxicity, the probability of survival was visualised using Kaplan–Meier survival curves, and the rate of survival analysed using the log-rank Mantel-Cox test. To test for differences in the potency of the knockdown effects, a Kruskal–Wallis test followed by Dunn’s pairwise post hoc comparison was used based on the 30-min timepoint as the earliest peak knockdown time point. In contrast, the comparison of mortality as a result of boric acid alone and that of boric acid in combination with either nicotine or caffeine was analysed using the Mann–Whitney *U*-test based on data at the 24-h timepoint. All of the analyses and generated graphs were done using GraphPad Prism software (GraphPad Prism, v. 8.0.0; GraphPad Software, San Diego, CA) and variance is indicated on the graphs, where appropriate.

## Results

### ATP overrides the aversive effect of antifeedant and toxicant compounds

To assess whether the mosquitoes were able to detect the antifeedant and toxicant compounds, no-choice feeding assays were performed, which demonstrated that all tastants elicited an aversive response when combined with ATP at EC_50_ (0.072 mM; Fig. 1a, b). In contrast, when tested at the same concentration, the antifeedant and toxicant compounds in combination with ATP at EC_max_ (0.6 mM) elicited no aversive response (Fig. 1b), demonstrating that ATP is able to override the aversive effect of these tastants. A comparison of the proportion of females engorging in response to a range of concentrations of antifeedant and toxicant compounds together with ATP (EC_max_) demonstrated an overall significant difference in sensitivity to various antifeedant and toxicant compounds [*F*_(7,212)_ = 30.54, *P* < 0.001; Fig. 1c]. More specifically, the antifeedants and toxicants were ranked with respect to female mosquito sensitivity as follows: DEET (EC_50_ = 0.39 mM) = quinine [EC_50_ = 0.23 mM; *F*_(1, 61)_ = 1.19, *P* = 0.28] < lobeline [EC_50_ = 1.14 mM; *F*_(1, 61)_ = 23.03, *P* < 0.001] = nicotine (EC_50_ = 1.25 mM; *F* = 0.21, *P* = 0.65) < capsaicin (EC_50_ = 3.07 mM; *F* = 57.15, *P* < 0.001) < caffeine (EC_50_ = 9.34 mM; *F* = 51.37, *P* < 0.001) < boric acid (EC_50_ = 321.80 mM; *F* = 16.50, *P* < 0.001) < propylene glycol (EC_50_ = 1429 mM; *F* = 8.11, *P* = 0.0070) (Fig. 1c).

The observed volume of antifeedant- and toxicant-laden diets imbibed due to ATP phagostimulation was higher (stage iv–v) than that observed in the sugar-induced feeding (stage ii–iii; Fig. 1a). However, the volume of nicotine imbibed due to ATP-induced feeding was lower than that of the other antifeedants or toxicants tested, ranking on average as fed (stages ii–iii) compared to engorged (stages iv–v), respectively (Fig. 1a). In general, the females that fed on the antifeedant- and toxicant-laden meals displayed similar windows of dynamic response to the antifeedant and toxicant compounds, as indicated by similar slopes of the linear parts of the curves (*F* = 2.87, *P* = 0.0070; Fig. 1c). While similar, the slopes associated with feeding on DEET (slope = − 0.92) and propylene glycol [slope = − 1.21; *F*_(1, 44)_ = 0.39, *P* = 0.53] demonstrated wider dynamic windows for these antifeedants and toxicants when compared to the rest of the compounds, which shared similar dynamic windows [*F*_(5, 168)_ = 0.49, *P* = 0.80; Fig. 1c].

### Feeding on toxicant-laced diets elicits toxic effects

**Fig. 2a–h:**
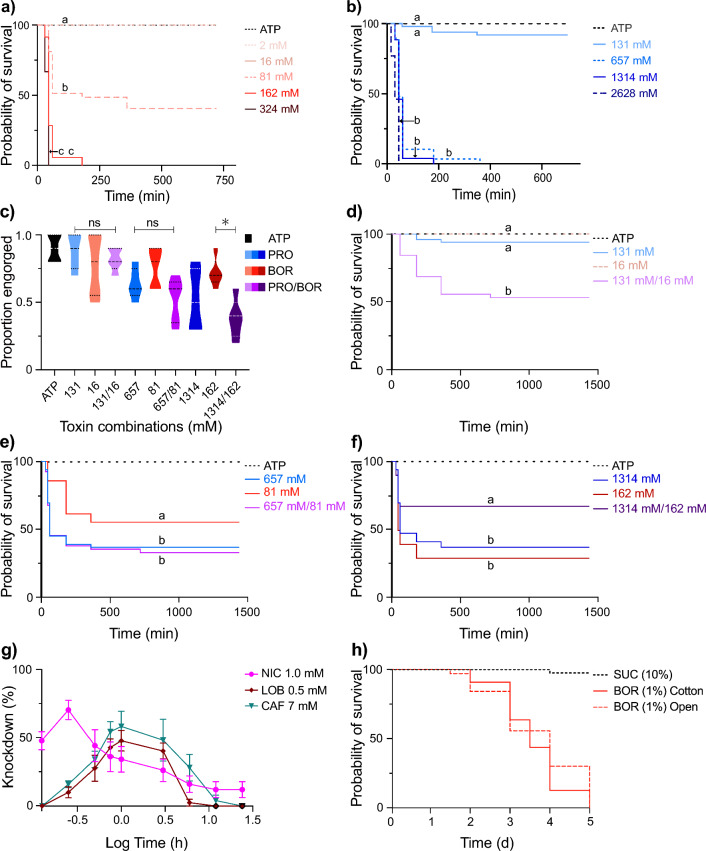
ATP-induced feeding elicits rapid antifeedant- and toxicant-associated toxic effects in *Aedes aegypti*. Kaplan–Meier probability of survival curves for **a** ATP and boric acid (*BOR*) and **b** ATP and PRO in the engorged individuals. **c** Comparisons of the proportion of engorged individuals on ATP-induced feeding on BOR and PRO, and combinations thereof, over various concentrations. Comparison of Kaplan–Meier survival curves in response to ATP-induced feeding on BOR and PRO, and combinations thereof, over the low (**d**), middle (**e**) and high (**f**) concentrations. **g** Proportion of mosquitoes knocked down at the most potent concentrations of nicotine (*NIC*), caffeine (*CAF*) and lobeline (*LOB*) following exposure to ATP-containing meals. **h** The probability of survival of sugar-induced feeding on BOR in open access (dotted line) and cotton wick (solid line) exposure assays. A log-rank (Mantel-Cox) statistical test was used to analyse the probability of survival, while a Kruskal–Wallis test was used to analyse the proportion of engorged mosquitoes; * *P* < 0.001. The colour intensities represent the increase in concentrations, while the lowercase letters indicate significant differences among the individual concentrations tested and the combinations thereof. For other abbreviations, see Fig. 1

ATP-induced feeding on boric acid and propylene glycol elicited rapid lethal effects in individuals that engorged (Fig. 2a, b), as well as in those that had fed, but not engorged, on the diets (Additional file 2: Fig. S1a, b). Boric acid (*χ*^2^ = 247.50, *df* = 5, *P* < 0.001) and propylene glycol (*χ*^2^ = 163.00, *df* = 4, *P* < 0.001) elicited significant mortality in engorged individuals in a dose-dependent manner, with maximum mortality observed within the first 3 h for the two and three highest doses tested of the respective toxicants (Fig. 2a, b). Among the total individuals exposed to the highest concentration of propylene glycol, mortality was observed in those that had engorged as well as in those that imbibed less than a complete meal (Additional file 2: Fig. S1b).

The highest doses of boric acid and propylene glycol that did not result in a reduction in the proportion of mosquitoes engorging (boric acid, 16 mM; propylene glycol, 131 mM; Fig. 2c) caused no significant effect on mortality alone, but when combined, elicited a synergistic effect on mortality (Fig. 2d). The second lowest combined doses, eliciting both engorging and feeding (boric acid, 81 mM; propylene glycol, 657 mM), elicited an effect similar to that of propylene glycol alone (propylene glycol, 657 mM; Fig. 2e; Additional file 2: Fig. S1c), while at the highest doses tested (boric acid, 162 mM; propylene glycol, 1314 mM), mortality reduced with the combined diet compared with the individual toxicants (Fig. 2f), likely due to the reduced diet intake (Fig. 2c).

### ATP-induced feeding on antifeedants and boric acid diets elicits knockdown toxic effects

In addition to the observed lethal effects, knockdown effects were observed in response to feeding on nicotine, lobeline and caffeine in a dose-dependent manner (Fig. 2g; Additional file 2: Fig. S1d–f). Nicotine elicited a more rapid knockdown effect, peaking after 30 min, compared with lobeline and caffeine (*H* = 10.76, *df* = 2, *P* < 0.001; Fig. 2g). Dunn’s post hoc comparison indicated a significantly higher knockdown effect for nicotine than caffeine (*Z* = 2.20, *P* = 0.030) or lobeline (*Z* = 3.21, *P* = 0.0010), whereas the knockdown effects of caffeine and lobeline were not significantly different (*Z* = 1.010, *P* = 0.31; Fig. 2g).

To assess the possible synergistic effect of nicotine and boric acid on mortality, mosquitoes were fed on combined diets, which demonstrated that the reduction in engorgement elicited by nicotine at the two highest doses tested resulted in reduced mortality in the combined diets compared to that induced by boric acid alone (Additional file 3: Fig. S2a, b). The lowest doses tested elicited no difference in engorgement and no mortality in response to the combined diet (Additional file 3: Fig. S2a, b). To assess the potentiation effect of caffeine on mosquitoes engorging on boric acid, 5 mM caffeine, the concentration eliciting the highest level of engorgement alone, was used together with various concentrations of boric acid in the toxicity assay. There was no increase in the proportion of mosquitoes feeding [*F*_(2, 30)_ = 1.53, *P* = 0.23; Additional file 3: Fig. S2c], nor was there an increase in the mortality within the corresponding combinations (*χ*^2^ = 9.5, *df* = 5, *P* = 0.091; Additional file 3: Fig. S2d–f), when combined caffeine and boric acid diets were analysed.

### Sucrose-induced feeding on boric acid-containing diets elicits slow lethal effects

To compare the mortality rates in response to ATP- and sugar-induced diets, three modes of sugar meal delivery were assessed. While membrane feeding did not elicit sufficient numbers of fed mosquitoes within 30 min to compare with ATP-fed individuals, open source and cotton wick feeding ad libitum resulted in diet uptake and significant mortality (comparison between sucrose plus boric acid vs. sucrose alone, *χ*^2^ = 108, *df* = 2, *P* < 0.001; comparison between open source vs. cotton wick feeding, *χ*^2^ = 2.51, *df* = 1, *P* = 0.11; Fig. 2h). The maximum mortality in response to feeding on boric acid-laced sugar diets occurred at or before 5 days (Fig. 2h).

### Diet destination is modulated by the type of phagostimulant

**Fig. 3:**
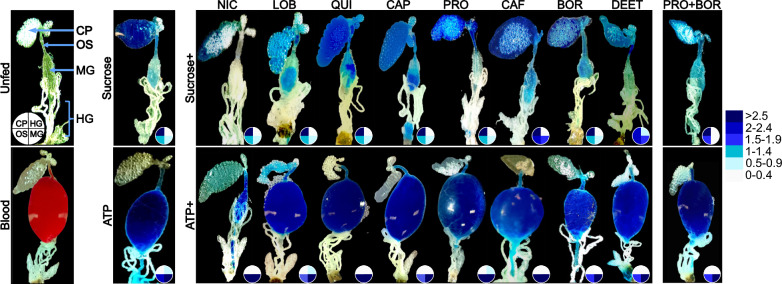
ATP and sucrose variably modulate the final destination of the meal. Exemplar micrographs of gut dissections of *Aedes aegypti* demonstrate the predominant destination of antifeedant- and toxicant-laced meals [NIC, LOB, quinine (*QUI*), capsaicin (*CAP*), CAF, BOR] to the crop (*CP*), oesophagus (*OS*), midgut (*MG*) and hindgut (*HG*). The control gut dissections for unfed, sucrose-, blood- and ATP-fed mosquitoes are presented in the four leftmost panels. The guts of mosquitoes fed on antifeedant- and toxicant-laden meals induced by either sucrose or ATP are shown in the upper and lower panels, respectively. The pie charts represent a semi-quantitative indication of the distribution of the meals to the four main gut regions. The colour intensities in the pie chart (scale to the right) visually rank the average intensity of the blue dye in the gut regions on a scale of 0 (lowest) to 3 (highest (*n* = 10). For other abbreviations, see Figs. 1 and 2

To assess whether ATP and sucrose modulate the destination of the antifeedant and toxicant diet in the mosquito, gut dissections were performed. In the mosquitoes that were induced to feed on blood or ATP-containing diets the meals were predominantly directed to the midgut, whereas in those induced to feed on sucrose-containing diets the meals were predominantly directed to the crop (Fig. 3). It is noteworthy that caffeine induced an increase in the volume imbibed when combined with sucrose (stage iv) compared to the other diets (stage ii, iii), while feeding on both caffeine and DEET, in combination with sucrose, led to variable distribution of the meal to different destinations in the gut (Fig. 3).

## Discussion

The ATSB technology used in the control of mosquito vectors relies on sugar as the phagostimulant for the toxin-laden meals. A general food substrate for many non-target organisms, sugar induces intermittent feeding by mosquitoes [24], requiring multiple visits to the control tool, which results in a low rate of toxicity [5]. In this study, we evidenced that ATP is a potent model-alternative phagostimulant to sucrose, overriding the aversive effect of a range of structurally divergent antifeedant and toxicant compounds in a dose-dependent manner. The ATP-induced feeding on a toxic diet predominantly directed the meal to the midgut and rapidly elicited both knockdown and lethal effects, contrary to the slower acting sugar-induced toxicity (this study; [5, 17]). The combination of ATP and binary mixtures of toxic compounds resulted in either synergistic or additive lethal effects at low concentrations. In sum, we identified a novel and more effective pathway for delivering toxic and/or modifying agents in mosquito vector control tools.

Feeding on ATP-containing meals induces rapid ingestion of high volumes of the meal that are directed to the midgut, as part of a chain of reflexes initiated during host seeking [24], while feeding on sucrose induces a slower ingestion of smaller meal volumes, which are directed predominantly to the crop [15, 25–28]. This difference in feeding strategy likely relates to the risk of assault associated with host-seeking and blood-feeding [29, 30] compared to feeding on nectar resources. Both ATP and sucrose dose dependently override the aversive response induced by antifeedant and toxicant compounds, with ATP driving the meal uptake at a more rapid rate and to a greater volume than sucrose (this study; [15, 26]). The behavioural response to the antifeedant and toxicant compounds suggests differential sensitivity of the pathways detecting aversive compounds, similar to that observed in *Drosophila*, in which antifeedant and toxicant compounds can activate antifeedant- and toxicant-sensitive receptors and/or suppress the response of sugar-sensitive neurons [31, 32]. Behavioural evidence in mosquitoes (this study; [13, 33]) suggests that chemosensory neurons in sensilla on the labrum, or in the cibarium, differentially detect antifeedants and toxicants. However, whether antifeedant compounds modulate the response of sensory neurons in these sensilla in mosquitoes, or act through a dedicated pathway, is currently unknown. As with other flower-visiting insects, mosquitoes have evolved the ability to detect aversive compounds as a response to their presumed toxicity in nectar sources [32, 34, 35]. Similarly, there appears to be a selection pressure to detect antifeedant compounds in blood (this study; [13, 33]), as evidenced by the ability of obligate haematophagous animals to maintain antifeedant-sensitive receptors over an evolutionary timescale [36].

The risk associated with naturally occurring alkaloids likely explains the higher sensitivity to these tastants compared to boric acid, the most common lethal agent [5], and the synthetic compound propylene glycol, a potentially safer toxicant [17, 37] for use in ATSBs. Of the alkaloids tested in this study, nicotine, lobeline and caffeine elicited a knockdown effect, likely by acting as agonists of the nicotinic acetylcholine receptors [38, 39]. While the cholinergic antagonist quinine [40] had no effect in *Ae. aegypti* (this study; [13]), in *Anopheles gambiae,* blood meals containing quinine induced knockdown effects [33], likely due to differences in sensitivity to quinine. In contrast, the mechanisms of action of boric acid and the breakdown products of propylene glycol are direct, with the former acting as a stomach poison in the midgut of mosquitoes, disrupting the gut epithelium, affecting metabolism, and potentially acting as a neural toxin [41]. While the ability to detect and evade antifeedant- or toxicant-containing meals is an innate response, this can be overridden by the use of a phagostimulant, which in turn influences the meal destination and the rate of toxicity.

ATP-induced feeding elicited a more rapid toxic effect compared to sucrose-induced feeding (this study; [17–19, 23, 42]), as a result of directing the meals containing the toxic agents almost exclusively to the midgut, as opposed to the crop. The destination of the meals was not influenced by the type of the antifeedant or toxicant compound contained within, with two notable exceptions, caffeine and DEET, when combined with sucrose. This suggests that the detection of select antifeedants and toxicants has the potential to affect diet destination. Caffeine combined with boric acid was predicted to potentiate the lethal effects of boric acid when feeding was induced by ATP; however, this was not observed in the present study. In contrast, combinations of low doses of boric acid and propylene glycol significantly and synergistically enhanced the lethal effect. Combinations of the most potent knockdown-inducing compound, i.e. nicotine, with boric acid, did not increase the lethal effect of the meal, likely due to nicotine regulating the ingestion of low diet volumes at the high concentrations. Overall, the available results suggest that phagostimulants triggering feeding via the ATP-pathway may be used to improve the efficacy and efficiency of the technology in the control of disease vectors.

ATP as a model phagostimulant in taste-based control tools provides additional advantages compared with sucrose. In principle, not only would ATP agonists increase selectivity for haematophagous vectors, these would also include obligate blood feeders [8] that are currently not targeted by sugar-based control tools. Resistance is a major factor limiting the usefulness of currently available vector control tools [43–45]. Moreover, resistance to glucose, caused by the misexpression of a sugar receptor in the aversive sensory neuron, has led to a loss of efficacy of ATSBs used to control cockroaches [7]. The evolutionary cost of a mutation in the ATP-detection pathway would detrimentally affect vector fitness and is thus highly unlikely to be a “selected for” trait. Taken together, the advantages associated with ATP as a phagostimulant strongly suggest that its thermostable agonists represent a set of alternative phagostimulants to sucrose for future oral-based vector control technologies. Such technologies would allow for the oral delivery of other vector-modifying agents, including biological material (e.g. *Bacillus thuringiensis* toxins), genetic material (e.g. double-stranded RNA) and chemical agents [5, 46–48].

## Conclusions

*Aedes aegypti*, used as a representative haematophagous vector, responds reflexively to ATP and demonstrates variable sensitivities to ATP-laced antifeedant and toxicant meals. ATP non-specifically overrides the aversive effect of a range of structurally divergent antifeedants and toxicants in a dose-dependent manner. Based on the combined aspects of ATP-driven feeding responses, i.e. reflexive engorgement, high meal volume and midgut diet destination, ATP serves as a model alternative phagostimulant delivering more efficient toxic effects than sucrose when used in available ATSBs, which non-selectively affects off-target organisms, and does not target obligate blood-feeding vectors. Being an ephemeral molecule, rapidly degrading at room temperature, further studies are required to identify structurally related, stable analogues of ATP, such as the non-hydrolysable ATP analogues adenylyl imidodiphosphate and adenylyl methylene diphosphate [49], from among the currently known and commercially available ATP analogues [50, 51]. Such analogues may allow for the use of more lethal and eco-friendly toxic compounds, which can be used individually or synergistically, in future attractive toxic bait technologies. Further still, to ascertain the wide application of ATP analogue-based toxic baits, the stable analogues should be examined with a wider array of representative haematophagous vectors under both laboratory and field conditions. For this purpose, the workflow presented in this study is ideal, and is amenable to the recently developed bait stations designed to prevent off-target species feeding [52].

### Supplementary Information

**Additional file 1. Table S1**: The concentrations (millimolar; mM) of combined antifeedants and toxicants and their corresponding percentages. The tabulation indicates combinations of propylene glycol (*PRO*) and boric acid (*BOR*), BOR and nicotine (*NIC*), as well as BOR and caffeine (*CAF*).

**Additional file 2. Figure S1**: Toxic effects of adenosine triphosphate (ATP)-induced feeding on toxicant- and antifeedant-laced meals by *Aedes aegypti*. The probability of survival among all of the individuals having been exposed to diets containing ATP (0.6 mM) together with **a** BOR, **b** PRO and **c** a combination of the two toxins. The proportion of individuals knocked down in response to having been exposed to diets containing ATP and **d** nicotine, **e** lobeline and **f** caffeine. Different lowercase letters indicate significant difference among treatments and concentrations.

**Additional file 3. Figure S2**: Evaluation of the potentiation effect of combing antifeedant compounds inducing knockdown with BOR. **a** The proportion of engorged individuals on BOR and NIC, as well as combinations thereof. The gradations in colour indicate the corresponding increase in concentrations. **b** The mortality rate in response to these treatments after 24 h. **a**, **b** *P < 0.05, **P < 0.001, ns non-significant. **c** The proportion of engorgement in individuals induced to feed on meals laden with BOR or BOR and CAF. Kaplan–Meier survival curves for the corresponding mortality rates of BOR as well as BOR in a 5-mM CAF background for the low (**d**), average (**e**) and high (**f**) concentrations. Different lowercase letters indicate significant difference among treatments and concentrations.

## Data Availability

All data generated or analysed during this study are included in this published article and Additional files 1–3.

## Abbreviations

ATP: Adenosine triphosphate
ATSB: Attractive toxic sugar bait
DEET: *N*,*N*-diethyl-meta-toluamide
