# Supplementary figures and images for: Adenosine triphosphate overrides the aversive effect of antifeedants and toxicants: a model alternative phagostimulant for sugar-based vector control tools
Source: Parasit Vectors. 2023 Nov 14;16:416. doi: 10.1186/s13071-023-06039-x (PMC10647091; doi:10.1186/s13071-023-06039-x)

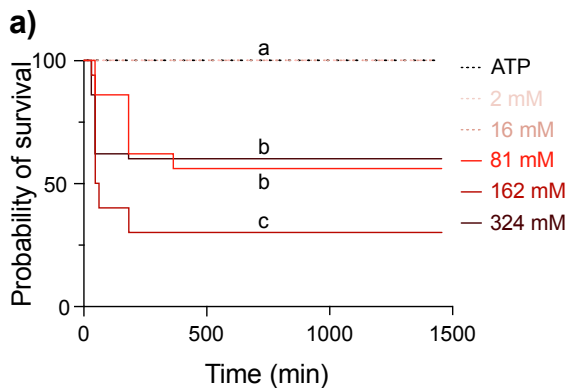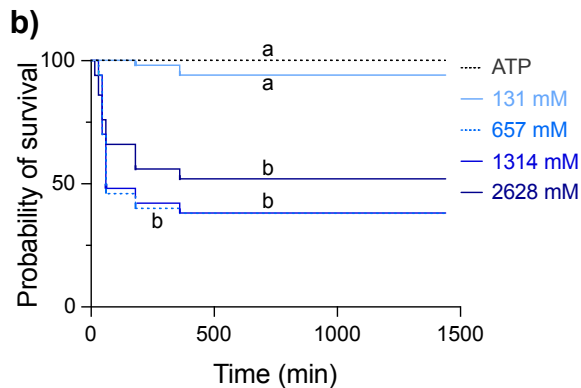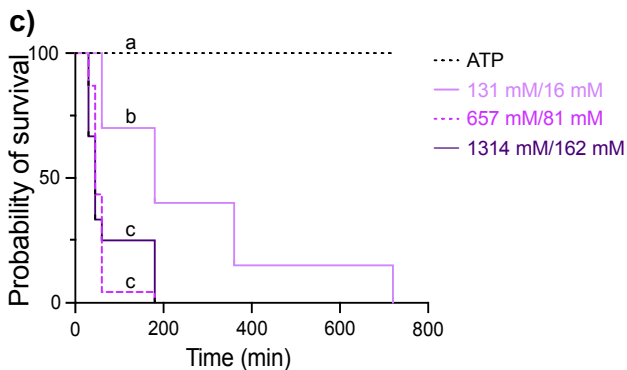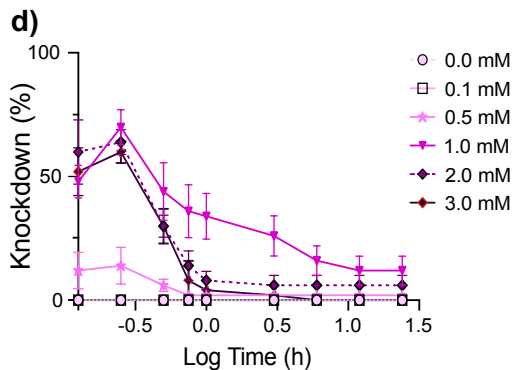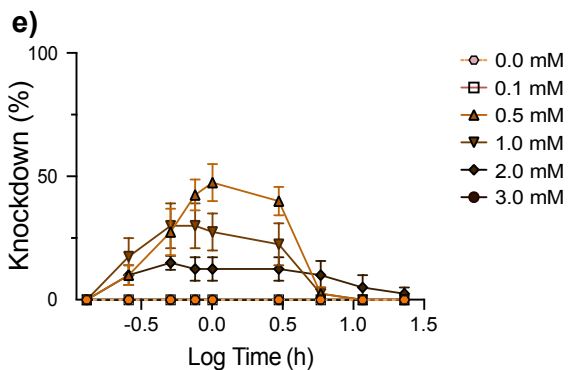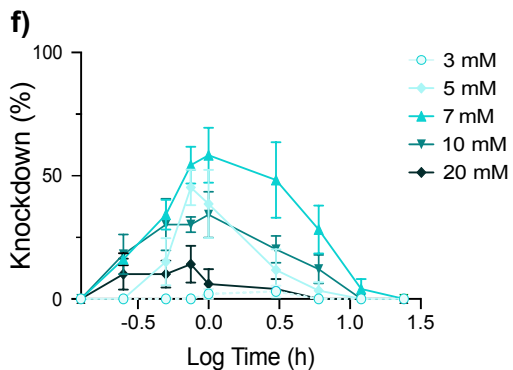

Supplement: Supplementary file 2 — Additional file 2. Figure S1: Toxic effects of adenosine triphosphate (ATP)-induced feeding on toxicant- and antifeedant-laced meals by Aedes aegypti. The probability of survival among all of the individuals having been exposed to diets containing ATP (0.6 mM) together with a BOR, b PRO and c a combination of the two toxins. The proportion of individuals knocked down in response to having been exposed to diets containing ATP and d nicotine, e lobeline and f caffeine. Different lowercase letters indicate significant difference among treatments and concentrations. [file 13071_2023_6039_MOESM2_ESM.pdf]

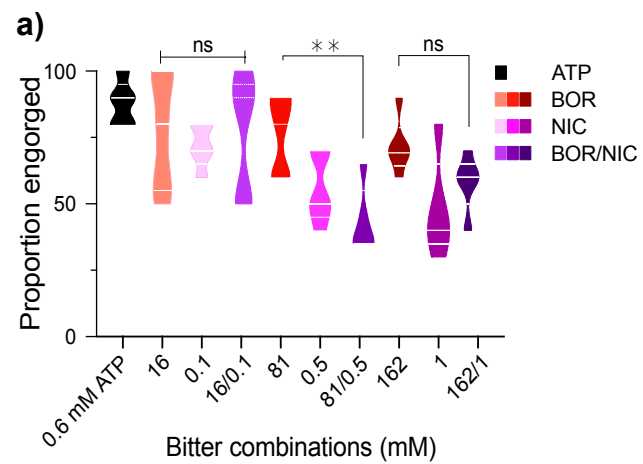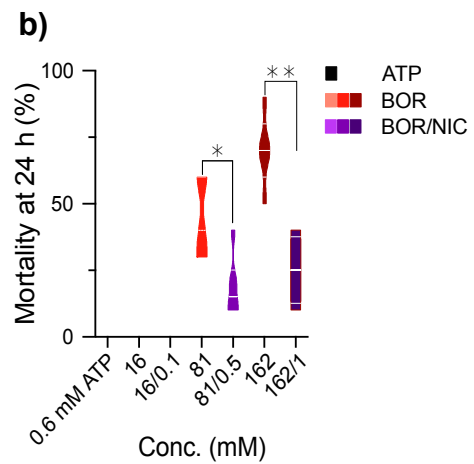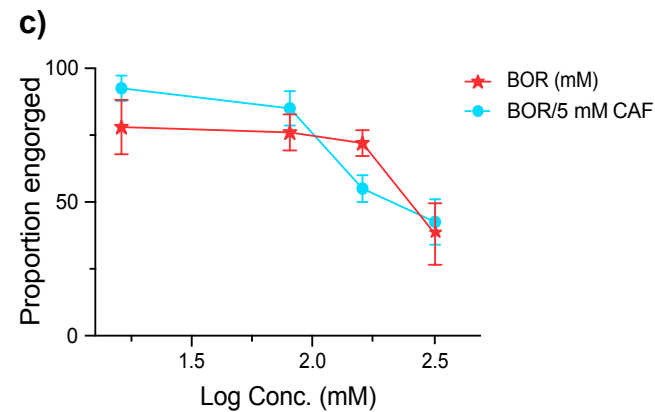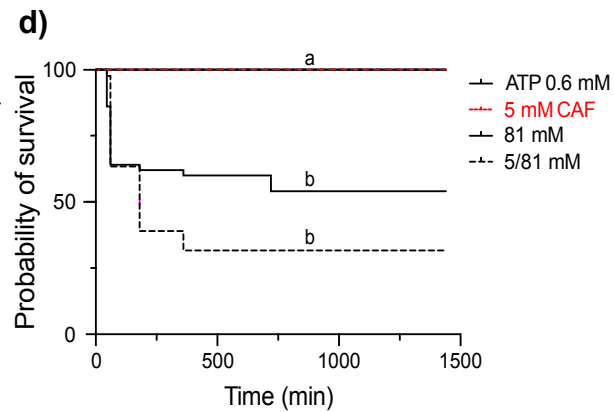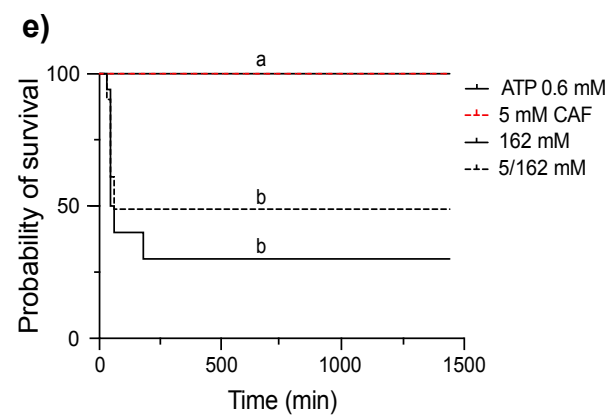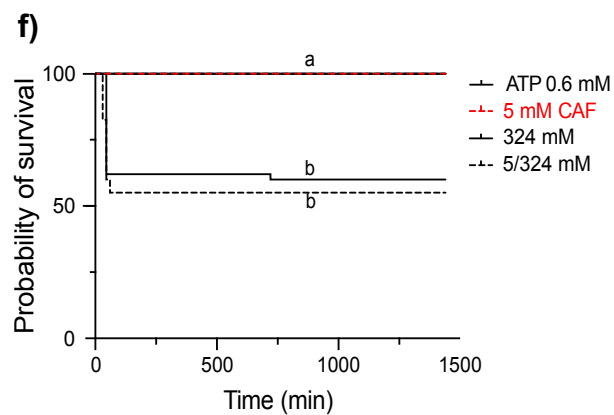

Supplement: Supplementary file 3 — Additional file 3. Figure S2: Evaluation of the potentiation effect of combing antifeedant compounds inducing knockdown with BOR. a The proportion of engorged individuals on BOR and NIC, as well as combinations thereof. The gradations in colour indicate the corresponding increase in concentrations. b The mortality rate in response to these treatments after 24 h. a, b *P < 0.05, **P < 0.001, ns non-significant. c The proportion of engorgement in individuals induced to feed on meals laden with BOR or BOR and CAF. Kaplan–Meier survival curves for the corresponding mortality rates of BOR as well as BOR in a 5-mM CAF background for the low (d), average (e) and high (f) concentrations. Different lowercase letters indicate significant difference among treatments and concentrations. [file 13071_2023_6039_MOESM3_ESM.pdf]
